# Neuroimaging Markers of Chronic Eye Diseases and Their Application Values

**DOI:** 10.3389/fneur.2022.854605

**Published:** 2022-06-14

**Authors:** Chen-Yu Yu, Rong Huang, Shi-Qi Li, Yi Shao

**Affiliations:** Department of Ophthalmology, Jiangxi Province Ocular Disease Clinical Research Center, The First Affiliated Hospital of Nanchang University, Nanchang, China

**Keywords:** magnetic resonance imaging, chronic eye diseases, markers, neurology, application

## Abstract

In recent years, the impact of various chronic eye diseases on quality of life has become increasingly apparent. Therefore, it is particularly important to control the progress of chronic diseases at an early stage. Many studies have used neuroimaging methods to explore the effects of chronic eye diseases on the brain, and to identify changes in brain function that may act as markers for early diagnosis and treatment. This article reviews the clinical application of different techniques of functional magnetic resonance imaging in chronic eye diseases.

## Application of ALFF in Chronic Eye DiseaseS

As a resting-state functional magnetic resonance imaging (MRI) analysis tool, the amplitude of low-frequency fluctuation (ALFF) has high accuracy and repeatability and does not require definition of regions of interest (ROI) in advance. By calculating the root mean square of the blood oxygen level dependent (BOLD) signal power spectrum at low frequencies (0.01HZ−0.08HZ), the neuronal activity in different brain regions is expressed and is an indicator of spontaneous neuronal activity (1). The value of ALFF technology in detecting brain neuron activity has been confirmed in previous experiments (2). Changes in ALFF value reflect disease progression, in part and are widely used in the diagnosis and monitoring of eye diseases. ALFF markers for chronic eye disease are shown in Table 1, where the middle frontal gyrus (MFG) features prominently. However, changes in ALFF values at the MFG have implications that vary with disease. For example, in patients with monocular blindness (5) and neovascular glaucoma (10), ALFF changes at the MFG are related to visual perception impairment or compensation, while similar changes in diabetic vitreous hemorrhage (9) indicate visual motor disorder, and in strabismic amblyopia (7), diabetic retinopathy and nephropathy (8) they may indicate a tendency toward anxiety and depression.

**Table 1:** Application of ALFF in chronic eye diseases.

**Disease**	**Year**	**Increased ALFF values**	**Decreased ALFF values**
High myopia (3)	2016	BMCC, LposCG, LpreCU/IPL	RITG/MTG, LMTG, LIFG/PT, RIFG/PT/IS, RMFG, RIPL
Diabetic retinopathy (4)	2016	BOG, RLG, preCU	RP/ACL, RPG, RFG, RSTG, RIPG, RAG
Monocular blindness (5)	2016	RMFG, LMFG, LSMG	LCAL, RMG, RC, LPCG/PCL
Congenital comitant strabismus (6)	2016	BCPL, LAG	BMFG
Strabismus with amblyopia (7)	2018	RSFG, RPC, LC, BPCG	LCPL, LT, RT, LMFG
Diabetic retinopathy and nephropathy (8)	2019	BCPL, LITG	BMFG, RSTG, RMFG, LMFG, BP, LIPL
Vitreous hemorrhage (9)	2020	RCPL, LCPL, LCPL/LLG, BSFG/LPG	RMFG, RIFG, RMFG/LAC, RSFG, RSFG/MFG, LMFG
Neovascular glaucoma (10)	2020	RSFG, LMFG	RC, RMOG, RP, LCG, LMFG

*RITG/MTG, right inferior and middle temporal gyrus; LMTG,left middle temporal gyrus; LIFG/PT, left Inferior frontal gyrus/putamen; RIFG/PT/IS, right inferior frontal gyrus/putamen/insula; RMFG, right middle frontal gyrus; RIPL, right inferior parietal lobule; BMCC, bilateral midcingulate cortex; LposCG, left postcentral gyrus; LpreCU/IPL, left precuneus/inferior parietal lobule; BOG, bilateral occipital gyrus; RLG, right lingual gyrus; RP/ACL, right posterior/anterior cerebellar lobe; RPG, right parahippocampal gyrus; RFG, right fusiform gyrus; RSTG, right superior temporal gyrus; RIPG, right inferior parietal gyrus; RAG, right angular gyrus; LAG, left angular gyrus; LSMG, left supramarginal gyrus; RC, right cuneus; LC, left cuneus; LCAL, left cerebellum anterior lobe; LPCG/PCL, left precentral gyrus/paracentral lobule; BCPL, bilateral cerebellum posterior lobe; RSFG, right superior frontal gyrus; RPC, right precuneus; BPCG, bilateral precentral gyrus; LCPL, left cerebellum posterior lobe; LT, left thalamus; RT, right thalamus; LITG, left Inferior frontal gyrus; BP, bilateral precuneus; LLG, left lingual gyrus; LPG, left postcentral gyrus; LAC, left anterior cingulate;RP, right precuneus; RMOG, bilateral middle occipital gyrus; LCG, left cingulate gyrus*.

## Application of DC in Chronic Eye Diseases

Voxel-wise degree centrality (DC) is another commonly used technique in resting state functional MRI technology. It assesses functional connectivity within the brain by measuring the topological structure of brain functional connectors at voxel level, and provides the correlation between different nodes and the importance of each node in the network structure (11). The degree of direct functional connection between two nodes can be expressed by DC values. A high DC value indicates a higher degree of direct connection between the node and other nodes. A change in DC values indicates a change in connectivity between the node and the network and clearly shows the state of each node (12). This method can be used to find any changes in brain functional connectivity in chronic ocular disease, and the changemay be an important marker for disease detection. Chronic diseases and their corresponding DC value markers are shown in Table 2.

**Table 2:** Application of DC in chronic eye diseases.

**Disease**	**Year**	**Increased DC values**	**Decreased DC values**
Comitant exotropic strabismus (13)	2018	RSTG, BAC, LIPL	RCPL, RIFG, RMFG, RSPL/SI
Diabetic nephropathy and retinopathy (14)	2019	BP	RITG, LSG
Monocular blindness (15)	2019	LITG, BMFG	BC/V1/V2

*RSTG, right superior temporal gyrus; BAC, bilateral anterior cingulate; LIPL, left inferior parietal lobule; RCPL, right cerebellum posterior lobe; RMFG, right middle frontal gyrus; RSTG, right superior temporal gyrus; RIFG, right inferior frontal gyrus; BP, bilateral precuneus; LSG, left subcallosal gyrus; RITG, right inferior temporal gyrus; LITG, left inferior temporal gyrus; BMFG, Bilateral medial frontal gyrus; BC, Bilateral cuneus; V1, primary visual cortex, V2, secondary visual cortex*.

## Application of REHO in Chronic Eye Diseases

Regional homogeneity (ReHo) is a widely used analytical method in resting state MRI, and plays an important role in exploring changes in local synchronization of voxels in brain regions (16). The analysis of ReHo is based on the measurement of voxels, and is used to measure the synchronization of adjacent regions based on the similarity between the time series of a given voxel and its nearest adjacent time series (calculated by the Kendall consistency coefficient of BOLD time series) (17). The coherence and centrality of regional brain activity are closely related to the ReHo values. ReHo is usually calculated in the low frequency range (0.01Hz–0.1Hz). Like ALFF, ReHo does not need a priori definition of ROI and can provide information about the activity of the whole brain. ReHo has been widely used in various studies to explore the local synchronization of spontaneous fMRI signals. In addition, the ReHo method is used in many studies of chronic eye diseases. Altered ReHo values can be used as a marker to monitor progress of various diseases, as shown by Table 3. ReHo measures at the inferior temporal gyrus (ITG) and the right cuneus (RC) are common markersfor chronic eye disease. The significantly increased ReHo values of the ITG in concomitant exotropia (23) and monocular blindness (20) indicated the compensatory mechanism of visual function. In monocular blindness, the ReHo values of RC decrease significantly, indicating the interruption of synchronous neural activity. The RC also showed a decreased ReHo value in diabetic retinopathy (22), reflecting vision-related dysfunction in this area. In patients with anisometropic amblyopia (18), a clear increase in RC was related to the compensatory mechanism of eye movement.

**Table 3:** Application of ReHo in chronic eye diseases.

**Disease**	**Year**	**Increased ReHo values**	**Decreased ReHo values**
Anisometropic amblyopia (18)	2012	RINS/PUT, LSTG, LperCG, LFG, RMOG	RC, LMPC, LIFG, LC
Comitant strabismus (19)	2016	RITG/RFG/RCAL, RLG, BCG	_
Monocular blindness (20)	2017	RITG, RFMO, LPC/LP, LMFG	RRG, RC, RAC, RLOC
Strabismus and amblyopia (21)	2019	LLG, RMOG/RP, BAC, LMOG, BPG	LIFG
Diabetic retinopathy (22)	2019	RCPL, LCPL	RAC, RC, BP, LMFG
Constant exotropia (23)	2019	RV2	LBA47

*RINS/PUT, right insula and putamen; LSTG, left superior temporal gyrus; LperCG, left precentral gyrus; LFG, left fusiform gyrus; LMPC, left media prefrontal cortex; LIFG, left inferior frontal gyrus; RITG, right inferior temporal gyrus; LITG, left inferior temporal gyrus; RFG, right fusiform gyrus; RCAL, right cerebellum anterior lobe; RLG, right lingual gyrus; BCG, bilateral cingulate gyrus; RRG, right rectal gyrus; RFMO, right frontal middle orbital ; LPC, left posterior cingulate; LP, left precuneus; RP, right precuneus; BP, bilateral precuneus; LMFG, left middle frontal gyrus; RMFG, right middle frontal gyrus; RRG, right rectal gyrus; RC, right cuneus; RAC, right anterior cingulate; RLOC, right lateral occipital cortex; LLG, left lingual gyrus; RMOG, right middle occipital gyrus; LMOG, left middle occipital gyrus; BAC, bilateral anterior cingulate; RAC, right anterior cingulate; BPG, bilateral precentral gyrus; RCPL, right cerebellum posterior lobe; lCPL, left cerebellum posterior lobe; RV2, right secondary visual cortex; LBA47, left Brodmann area 47; RC, right cerebellum; LC, left cerebellum*.

## Application of FC in Chronic Eye Diseases

Functional connectivity (FC) is a seed-based or ROI-based functional connection, in which areas functionally related to activities in the seed area may be found (24). In seed-based analysis, cross-correlation is calculated between the time series of the seed and the rest of the brain to assess the activity of the selected brain region. Functional connections may be considered as brain areas which have been activated similarly, indicating that they have a similar role in brain functional activity. Physiologically, the relevant brain regions may not be directly connected by nerve fibers, but the connectivity matrix shows connection strength and range including indirect connections. Flexibility and sensitivity of this technique has resulted in it being widely used in the study of various brain functional diseases (25). The FC value indicates the intensity of activity and may be used to mark changes in brain functional activity caused by disease. Application of the FC method as a marker in brain functional activity of chronic ophthalmopathy is shown in Table 4.

**Table 4:** Application of FC in chronic eye diseases.

**Disease**	**Year**	**Increased FC values**	**Decreased FC values**
Anisometropic amblyopia (26)	2013	Lpost, LPL/MFG	BC, BIPL/AL, LMFL/PreG
Comitant exotropia (27)	2018	_	LLG/CPL, RMOG, LPreG/PG, RIPL/PG
Comitant Strabismus (28)	2019	PPVC, BA19, BA6	_
Neovascular Glaucoma (29)	2020	BMFG	LP, BC
Strabismus (30)	2021	BC, BC/RLG, LIOG, RMOG	LP/PG, RI/RO, LPG, BPL/PG/RPG/LPG

*LPostG, left postcentral gyrus; LPL/MFG, left paracentral lobule and the middle frontal gyrus; BC, bilateral cerebellum; BIPL/AL, bilateral inferior parietal lobe and the angular lobe; LMFL/PreG, left middle frontal lobe and the precentral gyrus; LLG/CPL, left lingual gyrus/cerebellum posterior lobe; RMOG, right middle occipital gyrus; LPreG/PG, left precentral gyrus/postcentral gyrus; RIPL/PG, right inferior parietal lobule/postcentral gyrus; BMFG, bilateral middle frontal gyrus; LP, left precuneus; BC, bilateral cuneus; RLG, right lingual gyrus; LIOG, left inferior occipital gyrus; RI/RO, right insula and rolandic operculum; RPG, right postcentral gyrus; PPVC, posterior primary visual cortex; BA, Brodmann area*.

## Application of VBM in Chronic Eye Diseases

Voxel-based morphometry (VBM) is an MRI whole-brain analysis technique for measuring density and volume changes of gray and white matter at the voxel level, and is used to enhance understanding of the anatomical structure of the brain (30). In contrast with some other resting MRI techniques, VBM has no preset region of interest, it detects changes in neural activity across all parts of the brain, and is an objective measure so is minimally influenced by subjective factors. The VBM approach filters the white and gray matter areas with statistically significant activity by comparing the processed MRI images (31), and can be used to detect pathological changes of brain function caused by disease. Changes of this kind have been found to accompany progression of many ophthalmic diseases, as shown in Table 5.

**Table 5:** Application of VBM in chronic eye diseases.

**Disease**	**Year**	**Altered WMV values**	**Altered GMV values**
Adult strabismus (32)	2004	_	OEF, PEF, PEF, SEF, PFC, SR
Amblyopia (33)	2005	_	C/PC, MPOJ, LPOJ, VTC
Comitant strabismus (34)	2017	LMTG, RMTG, RP, RPC	LMTP, LCPL, RPCC, LC, RPC
Monocular blindness (35)	2019	-_	RSM, RI, LI, RAC, LMOG, RIPL

*C/PC, calcarine and paracalcarine cortex; MPOJ, medial parietal-occipital junction; LPOJ, lateral parietal-occipital junction; VTC, ventral temporal cortex; WMV , white matter volume; GMV, grey matter volume; LMTG, left middle temporal gyrus; RMTG, right middle temporal gyrus; RP, right precuneus; RPC, right premotor cortex; LMTP, left middle temporal pole; LCPL, left cerebellum posterior lobe; RPCC, right posterior cingulate cortex; LC, left cuneus; RSM, right supra marginal; RI, right insular cortex; LI, left insular cortex; RAC, right anterior cingulate; LMOG, left middle occipital gyrus; RIPL, right inferior parietal lobe; OEF, occipital eye field; PEF, parietal eye field; FEF, frontal eye field; SEF, supplementary eye field; PFC, prefrontal cortex; SR, subcortical regions*.

## Application of VMHC in Chronic Eye Diseases

Voxel-mirrored homotopic connectivity (VMHC) is a new resting-state FC analysis method to measure the functional connection between hemispheres (36). This method can detect abnormal functional activity in local brain areas and changes of functional connection and synchronization of neural activity between corresponding regions in bilateral cerebral hemispheres at rest, which shows that the degree of separation of cerebral hemispheres is its outstanding advantage. The normal human brain generally has the characteristic of high synchronization of spontaneous nerve activity in homotopic regions between hemispheres. Many studies have confirmed that this characteristic may be generally destroyed in patients with chronic eye diseases, suggesting that hemispheric dysfunction plays an important role in brain dysfunction in patients with chronic eye diseases. Using VMHC, this loss of synchrony has been demonstrated in patients with diseases of this kind, as shown in Table 6.

**Table 6:** Application of VMHC in chronic eye diseases.

**Disease**	**Year**	**Increased VMHC values**	**Decreased VMHC values**
Early blindness (37)	2017	_	PVC, VAC, SAC
Monocular blindness (38)	2018	LI, LMFG	LC/C/LG, RC/C/LG, RPMC/PSC, RSPL
Comitant exotropia (39)	2018	STG, MFG	PreG, IPL, SPL
Diabetic retinopathy and nephropathy (40)	2020	_	BMTG, BMOG, BMFG

*LC/C/LG, left cuneus/calcarine/lingual gyrus; LI, left insula; LMFG, left middle frontal gyrus; RC/C/LG, right cuneus/calcarine/lingual gyrus; RPMC/PSC, right primary motor cortex (M1)/primary somatosensory cortex (S1); RSPL, right superior parietal lobule; BMFG, bilateral medial frontal gyrus; STG, superior temporal gyrus; PreG, precentral gyrus; IPL, inferior parietal lobule; SPL, superior parietal lobule; BMOG, bilateral middle occipital gyrus; BMTG, bilateral medial frontal gyrus; PVC, primary visual cortex; VAC, visual association cortex; SAC, somatosensory association cortex*.

## Application of Other Techniques in Chronic Eye Diseases

The fractional amplitude of low-frequency fluctuation (fALFF) provides a measure of inherent spontaneous brain activity (41). Measurement of fALFF values need to be carried out within a specified frequency range, and spontaneous brain activity can be expressed by the measurement of cerebral blood flow at the amplitude of low frequency oscillation (0.01–0.08 Hz) (42). The fALFF technique has the advantages of high sensitivity and specificity and is non-invasive, so it is widely used in brain functional activity imaging. Diffusion tensor imaging (DTI) is a widely used MRI method, which describes the diffusion direction of water as average diffusion coefficient (MD; diffusion within voxels) and fractional anisotropy (FA) (43). The overall extent of water diffusion may also be displayed. On the basis of eigenvalues (λ1, λ2, λ3) of diffusion tensor, scalar values ranging from 0 to 1 can be obtained. These are the FA values, which measure the overall directionality of water diffusion and the complexity of cytoskeleton structure, of great significance to the movement of water inside and outside of cells (44). Changes in direction of water diffusion help understand the pathological changes of myelin and other related brain tissues. On this basis, some studies have explored the application of DTI in eye diseases, and the value of DTI as a marker in the diagnosis of diseases. Arterial spin labeling (ASL) is a new technology developed on the basis of magnetic resonance perfusion imaging, which has high accuracy and is non-invasive. It can detect blood flow changes reflecting pathological changes in various regions of the brain. The ASL method has been successfully applied to trace the changes of local blood flow in eye diseases and is beneficial to disease diagnosis. Table 7 shows the application of fALFF, DTI and ASL methods in chronic eye diseases.

**Table 7:** Application of other techniques in chronic eye diseases.

**Disease**	**Year**	**fALFF**	
		**Increased fALFF values**	**Decreased fALFF values**
Monocular blindness (45)	2020	LP, RPI, LPI	LAC
Neovascular glaucoma (46)	2021	LP	RRO, LAC, RC
Disease	Year	DTI	
		Increased DTI values	Decreased DTI values
Amblyopia (47)	2013	PC	_
Comitant strabismus (48)	2016	LSTG	BMFG, RGP/B, BP
Disease	Year	ASL	
		Increased ASL values	Decreased ASL values
Comitant exophoria (49)	2018	RHP, BMFG/ACC, LIFG, RIFG, LSFG, BMCC, RMFG, RPL	_

*LP, left precuneus; RPI, right inferior parietal lobes; LPI, left inferior parietal lobes; LAC, left anterior cingulate; RRO, right rolandic operculum; RC, right caudate; LSTG, left superior temporal gyrus; BMFG, bilateral medial frontal gyrus; RGP/B, right globus pallidus/brainstem; BP, bilateral precuneus; RHP, right parahippocampal; ACC, anterior cingulate cortex; LIFG, left inferior frontal gyrus; RIFG, right inferior frontal gyrus; LSFG, left superior frontal gyrus; BMCC, bilateral medial cingulate cortex; RPL, right paracentral lobule; PC, prechiasmatic region*.

### Summary and Future Prospects

In summary, each magnetic resonance imaging technique has its own characteristics. To summarize the above, both ALFF and fALFF show regional differences in the brain, with high accuracy and repeatability, and do not need to pre-define regions of interest (ROI) (1), while fALFF makes improvements in noise reduction on the basis of ALFF (50); DC is more sensitive to showing the changes of connectivity in the brain network structure and the state changes of each node, so as to show the correlation of each network structure (11, 12); Both ReHo and FC can show the temporal distribution of voxels in brain functional regions (17, 25). ReHo focuses on describing the consistency within regions, while FC focuses on describing the synchronization between regions, but neither of them directly describes the intensity of brain activity in a certain region, that is, activity detection cannot be carried out. VMHC, as a new static FC analysis method, is more sensitive to the changes of functional synchronization between the two hemispheres of the brain (36). VBM focuses on exploring the changes of brain anatomy (51); DTI has irreplaceable advantages in understanding the complex cytoskeleton structure and other fine structures of the brain (43); ASL can track the changes of local blood flow in eye diseases and improve the accuracy of diagnosis.

In recent years, MRI has been increasingly widely used to explore disease-related changes in brain activity and functional connections. It provides a useful imaging index for understanding the mechanism and monitoring the progress of pathological changes in disease. Its role as a disease marker has been confirmed in many studies. Most chronic eye diseases are characterized by occult and chronic progression, which easily leads to missed diagnosis and misdiagnosis. Using MRI, changes in spontaneous brain activity which occur with eye diseases may be detected at an early stage and monitored, and then accurately locate the brain region where lesions occur, and combine clinical symptoms based on the consistent physiological functions of different brain regions to improve the accuracy of diagnosis, aiding both early diagnosis and treatment of chronic eye diseases. However, the application of magnetic resonance imaging as a marker in chronic eye diseases has some limitations, since physiological and hardware-related factors may affect the experimental results. In addition, due to overlapping functions of different brain regions, it may not be possible to accurately locate the affected areas of the diseased brain. Despite these limitations, MRI technology has great potential and scope to provide indicators of onset and progression of chronic eye diseases. With the continuous progress of technology, MRI technology will usher in a broader range of applications, increasing the scope for exploration of chronic eye diseases.

## Author Contributions

C-YY was responsible for the writing of the manuscript. RH was responsible for data analysis and the later submission. S-QL was responsible for collecting and screening the data. YS was responsible for the revision of the paper. All authors contributed to the article and approved the submitted version.

## Funding

The Central Government Guides Local Science and Technology Development Foundation (20211ZDG02003); Key Research Foundation of Jiangxi Province (No: 20181BBG70004); Excellent Talents Development Project of Jiangxi Province (20192BCBL23020); Natural Science Foundation of Jiangxi Province (20181BAB205034); Grassroots Health Appropriate Technology Spark Promotion Plan Project of Jiangxi Province (No: 20188003); Health Development Planning Commission Science Foundation of Jiangxi Province (No: 20201032); Health Development Planning Commission Science TCM Foundation of Jiangxi Province (No: 2018A060).

## Conflict of Interest

The authors declare that the research was conducted in the absence of any commercial or financial relationships that could be construed as a potential conflict of interest.

## Publisher's Note

All claims expressed in this article are solely those of the authors and do not necessarily represent those of their affiliated organizations, or those of the publisher, the editors and the reviewers. Any product that may be evaluated in this article, or claim that may be made by its manufacturer, is not guaranteed or endorsed by the publisher.
